# Reply to Mankowska et al. Comment on “Muth et al. Assessing Critical Flicker Fusion Frequency: Which Confounders? A Narrative Review. *Medicina* 2023, *59*, 800”

**DOI:** 10.3390/medicina59111929

**Published:** 2023-10-31

**Authors:** Thomas Muth, Jochen D. Schipke, Anne-Kathrin Brebeck, Sven Dreyer

**Affiliations:** 1 Institute of Occupational, Social, Environmental Medicine, Faculty of Medicine, Heinrich-Heine-University Düsseldorf, 40225 Düsseldorf, Germany; 2 Research Group Experimental Surgery, University Hospital Düsseldorf, 40225 Düsseldorf, Germany; 3 Artemis Augenklinik, 60314 Frankfurt, Germany; 4 Hyperbaric Oxygen Therapy, University Hospital Düsseldorf, 40225 Düsseldorf, Germany

First and foremost, we like to express our gratitude for the praise bestowed upon our narrative review [1], particularly as it comes from a group of such esteemed reputation [2].

Your question regarding the extent to which connectivity networks are involved in the processing of flicker light is entirely valid. However, addressing this question within the context of diving and hyperbaric medicine proves to be a formidable challenge. The reason for this challenge lies in the nature of field studies conducted in this environment. These studies involve measurements in a hyperbaric chamber or following a session in such a chamber, as well as measurements both above and below water. Assessing connectivity networks in this environment with sophisticated equipment appears to be feasible but with difficulties. Fortunately, these field studies have produced meaningful results, contributing, for instance, to the quantification of toxic effects associated with breathing gases.

You mention, as a peripheral aspect of critical-flicker-frequency threshold assessment, the complexity of various stimuli. We approach this notion with caution, particularly when it comes to solely discriminating between phases of light and dark.

On the other hand, we certainly concur that a multitude of questions remain unanswered. In this context, international standards would be highly desirable. These could encompass factors such as the size of the illuminated area, the colour of the light used, the distance between the observer and the test subject, and the question of whether frequency should increase from low to high values and should be increased manually or automatically.

Analogous regulations were published in 1996 by a task force in the field of heart rate variability after years of preparation [3]. Nevertheless, in the interim, quite a few new measures have been introduced, some of which have already been covered in review articles. This example serves to underscore the difficulty of initially establishing such a task force and the fact that, even if successful, its guidelines may only be adhered to for a limited duration.
